# Effectiveness of workplace health promotion programs for industrial workers: a systematic review

**DOI:** 10.1186/s12889-025-21365-8

**Published:** 2025-01-15

**Authors:** Sasha Javanmardi, Ludwig Rappelt, Sascha Zangenberg, Lars Heinke, Christian Baumgart, Daniel Niederer, Jürgen Freiwald

**Affiliations:** 1 Department of Movement and Training Science, University of Wuppertal, Wuppertal, Germany; 2 Department of Intervention Research in Exercise Training, German Sport University Cologne, Cologne, Germany; 3 Institute of Occupational, Social and Environmental Medicine, Goethe University Frankfurt, Frankfurt, Germany

**Keywords:** Occupational health, Systematic review, Methodological quality, Evidence-based practice, Blue-collar workers, Well-being

## Abstract

**Background:**

Workplace health promotion is essential for individual and organisational well-being and disease prevention, also in industrial workers. As the transfer of the evidence on the effectiveness of such programs into practice is limited due to scattered effects, the need for a consolidation of the available studies is given. The purpose of this systematic review was to synthesise the evidence on the effectiveness of workplace health promotion programs for industrial workers.

**Methods:**

An electronic literature search was conducted in PubMed, Cochrane Library, Web of Science™, Scopus, and EBSCOHost until July 26th 2023. Studies investigated industrial workers who performed manual labour for at least 20 h per week were included. They had to receive a workplace health promotion intervention under any control condition. Outcomes were workplace health interventions’ safety and corresponding health-related outcomes. The revised Cochrane risk-of-bias assessed the risk of bias (Rob 2) tool for randomised control trials (RCT) and cluster RCT. Quality assessment was performed using a modified Downs and Black Checklist.

**Results:**

Of the 25,555 studies initially identified, 39 were included. Generally, the mean quality of the studies was moderate, with most studies judged with a high overall risk of bias. Twenty-seven studies employed a behavioural approach, while one study adopted an organisational one. Ten studies utilised a multicomponent approach, and one intervention improved safety outcomes. The analysis of the results indicated an overall positive but heterogeneous effect across the different approaches.

**Conclusions:**

The studies included in this review provide evidence that workplace health promotion can be effective. However, the overall findings are inconclusive due to the high risk of bias. Therefore, the results should be interpreted cautiously. Despite the considerable amount of research conducted in this field, additional well-designed studies are needed to fully confirm the effectiveness and determine the most promising types of interventions for improving and maintaining industrial health.

## Background

Workplace health promotion programs aim to influence the workers’ health and productivity [1]. These programs encompass a range of activities designed to support and improve health among employees, including behavioural [2–7], organisational [8, 9], and safety interventions [10]. Their collective goal is to establish a healthy work environment and encourage positive health behaviours among employees [11]. There has been a notable surge in interest in recent years, indicating a growing recognition [12]. Given the substantial amount of time individuals devote to their jobs, the workplace offers a prime opportunity for health-enhancing initiatives [13]. Moreover, it can reach people of different socioeconomic statuses [14]. By addressing health concerns at both the individual and organisational levels, these programs aim to create a comprehensive approach to workplace health [11]. Recognising that investing in employee health can positively impact morale, productivity, absenteeism rates, and healthcare costs [15] underscores the importance of such programs in the modern workplace.

The health of blue-collar workers, including industrial workers, is a global concern [16–18]. Industrial workers are at a higher risk of developing chronic diseases or experiencing functional limitations owing to their low incomes, occupational status, and educational levels [19]. Previous studies have shown that the physical demands of work in an industrial setting can pose risks to workers’ health from a physical [18, 20–23] and psychological standpoint [24–26].

Despite the significant impact of occupational hazards on the health and well-being of industrial workers [27], comprehensive, evidence-based guidelines for preventing and managing these conditions are lacking. Research suggests that workplace health promotion effectively reaches out to workers, but it is still challenging to extend these efforts because of shift work and workers who have yet to report any health-related issues [28]. A growing number of systematic reviews and meta-analyses emerged in the field of workplace health promotion [29–31]. However, these analyses do not distinguish between the different populations. The evidence on the effectiveness of such programs in practice is further limited due to scattered effects. To the best of our knowledge, no review specifically addresses industrial workers. Conclusively, the purpose of our systematic review was to investigate the evidence on the effectiveness of workplace health promotion programs specifically for industrial workers. The objectives were to assess the effectiveness of workplace health promotion programs in improving health outcomes among industrial workers, identify effective interventions (behavioural, organisational, and safety), evaluate the quality of evidence, and determine the gaps in the current evidence.

## Methods

### Research Design and search strategy

This systematic review was pre-registered in PROSPERO (CRD42023445044). An electronic literature search was conducted using the following recommended databases: PubMed, Cochrane Library, Web of Science, Scopus, EMBASE(assessed via Cochrane), CINAHL (assessed via Cochrane), and EBSCOHost [32, 33]. The search strings and strategy used are listed in Table 1. The strings were adjusted for database-specific truncations, wildcards, and proximity operators. Articles were retrieved from the earliest possible date until July 26, 2023.

**Table 1:** Search strings and strategy

Variable	Search string
Physical activity	physical activity OR exercise OR training
Industrial workers	blue-collar workers OR factory workers OR manufacturing workers OR construction workers OR industrial workers
Health promotion	health OR health promotion OR occupational health promotion OR public health OR workplace health

To increase the sensitivity of the literature search, we expanded our search by manually searching grey literature and using Google Scholar to locate articles not found in major electronic databases. We searched for articles using the terms “workplace health promotion” combined with “blue-collar workers”, “industrial workers”, or “manufacturing workers”. The reference lists of the included studies were also checked to identify additional eligible studies. Preferred Reporting Items for Systematic Reviews and Meta-Analyses (PRISMA) were used to follow this process [34].

### Study eligibility criteria

The following criteria were established for screening titles and abstracts by three independent investigators:

- Randomised controlled trials (RCT); with parallel-group, cluster-randomised or crossover design.
- Articles written in English.
- Exclusion of animal studies and articles focused on injured population.
- No systematic reviews or meta-analyses.
- Interventions related to workplace health promotion and the safety of industrial workers.

### Inclusion and exclusion criteria

The search strings were determined using the Population, Intervention, Comparisons, and Outcomes (PICO) scheme. Included populations were industrial workers of both sexes aged 18–67 years who worked at least 20 h a week in industry or manufacturing and were active for at least six months. “Industrial workers” were defined as people who perform manual labour in a manufacturing setting [35]. Any interventions conducted in the workplace, either during working hours or shortly before or after work, were included with any passive and/or active controls and comparators. The main outcomes of interest were the different dimensions of occupational health and safety according to the WHO definition [11]. These outcomes included health-related outcomes, covering physical, mental, social, behavioural, and physiological domains, as well as other outcomes, such as work-related outcomes.

The identified studies were downloaded using a citation manager (Clarivate Analytics, EndNote 20.5, London, UK). After removing any duplicates, the studies were transferred to Rayyan [36]. Using the Prospero protocol, the eligibilities of the titles and abstracts were examined before the full text was evaluated. Two independent investigators (SJ and SZ) executed the methodological process, and a third investigator (LR) resolved any discrepancies.

### Evaluating the risk of bias

The revised Cochrane risk-of-bias tool (RoB2) was used to evaluate all included studies [37]. The tool addresses biases categorised into five domains: (1) the randomisation method, (2) deviations from predesignated interventions, (3) the absence of outcome data, (4) outcome measurement, and (5) selective reporting of findings. RoB2 for cluster RCTs was utilised, which additionally assesses the timing of identification or recruitment of participants as risk of bias [38]. An overall risk of bias domain was built based on the individual judgements; all outcomes with at least one high risk for a certain bias received an overall rating of “high risk”. Each domain is judged as ‘low risk of bias’, ‘some concerns’, or ‘high risk of bias’ [37]. Two investigators (SJ and LR) independently assessed the risk of bias. In case of disagreements, a third investigator (SZ) was consulted. The data was visualised with risk-of-bias VISualization in R [39].

### Methodological Study Quality Assessment

To evaluate scientific precision at the individual study level, a critical appraisal was independently performed by two investigators (SJ and SZ) using a modified version of the Downs and Black checklist [40]. This checklist for controlled studies includes 27 questions on internal validity, external validity, and power. Notably, item 27 was modified based on the presence of a power calculation, with a maximum score of 1 (a power analysis was performed) or 0 (absence of a power analysis). The highest attainable score on the checklist was 28 (instead of 32). The investigators’ results were compared, and potential disagreements were resolved by consensus. The final assessment of the quality of each study was calculated as a percentage, with a higher percentage reflecting greater quality. Consistent with previous recommendations [41], the ratings for each study were classified as low (≤ 33.3%), moderate (33.4–66.7%), or high (≥ 66.8%).

### Data extraction and evidence synthesis

One investigator (SJ) analysed the studies using the PICO scheme: (1) Participants = number of participants, sex, age, and working environment; (2) Intervention = type and dose (duration, time, intensity, frequency) of the intervention; (3) Comparators = type and dose (duration, time, intensity, frequency) of the control/comparator group; and (4) Outcomes = health-related outcomes. A second investigator (SZ) controlled the data extraction. Statistical interpretation of the results was provided only if the results were reported in the original study.

The interventions of the included studies were subsequently divided into categories of different workplace health promotion approaches or the category workplace health and safety [42]: Interventions to ensure workplace health and safety are based on department guidelines aimed at protecting workers from specific workplace risks (e.g., accidents, substance exposure, or noise) [42]. In the different workplace health promotion categories, the studies were classified into worker-directed approaches, called behavioural or work-directed approaches, called organisational approaches [43]. In addition, studies that used variable combinations were classified as multicomponent studies.

Due to the heterogeneity of outcomes and interventions of the studies, no meta-analysis could be performed. Instead, we provide a systematic narrative synthesis of the findings using the guidance of Popay and colleagues [44].

## Results

### Literature search

**Fig. 1:**
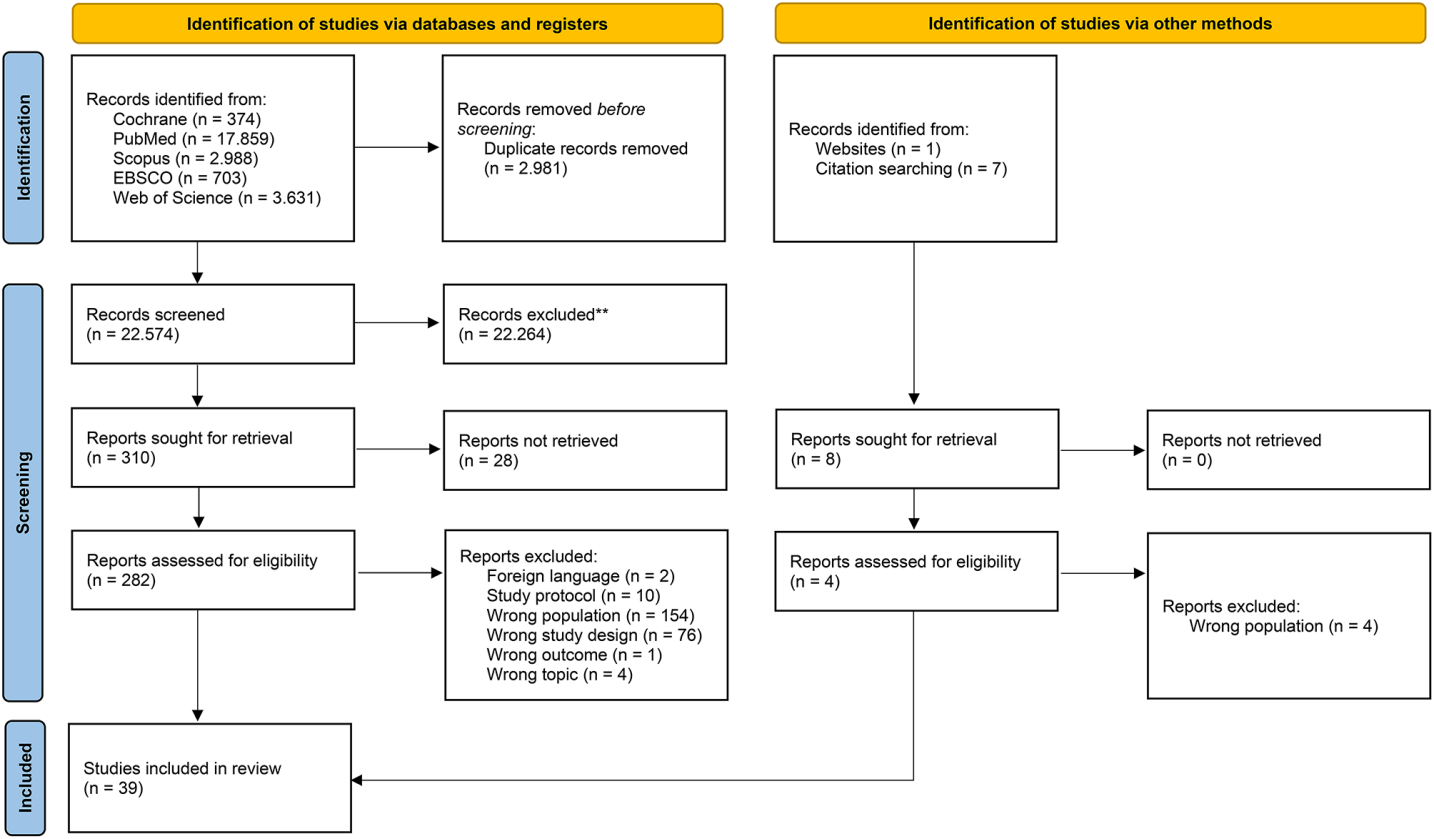
PRISMA flow diagram

Figure 1 provides the PRISMA flow diagram of the complete search process. Finally, 39 studies [45–83] were included in this systematic review.

### Description and characteristics of the included studies

The included studies are summarised in Table 2. All studies provided comprehensive data, eliminating the need to contact authors for further information. The studies were conducted across five continents and 18 countries, most of which were from Denmark (*n* = 9; [46, 48, 55, 56, 73–75, 81, 82]), the USA (*n* = 5; [47, 49, 59, 60, 78]), and Iran (*n* = 4; [45, 58, 64, 66]). The publication dates ranged between 1997 and 2023, with the majority (72%) published in the last decade.

**Table 2:** Characteristics of the included studies (*n* = 39)

Categories	Variables, number of studies, references
Type of intervention	Safety and health *n* = 1 [78] Organizational 1 [52] Behavior 27 [45, 46, 48, 50, 51, 53–55, 57–62, 65, 67–70, 72–76, 81–83] Multi-component 10 [47, 49, 56, 63, 64, 66, 71, 77, 79, 80]
Continent	North America 5 [47, 49, 59, 60, 78] South America 4 [52, 53, 65, 70] Europe 17 [46, 48, 51, 54–56, 62, 68, 69, 71, 73–75, 77, 81–83] Asia 12 [45, 50, 57, 58, 61, 64, 66, 67, 72, 76, 79, 80] Australia 1 [63]
Intervention duration	Acute interventions (< 6 weeks) 6 [45, 47, 50, 71, 76, 78] Sub-acute interventions (6–11 weeks) 11 [46, 51, 57, 58, 64, 66, 67, 72–75] Mid-term interventions (3–6 months) 15 [48, 49, 53–55, 60–63, 65, 68, 70, 77, 81, 82] Long-term interventions (> 6 months) 7 [52, 56, 59, 69, 79, 80, 83]
Health related outcomes	Physical domain 19 [48, 49, 51, 55–57, 60, 62, 63, 67–70, 72–74, 77, 81, 82] Mental domain 10 [46, 48, 50, 52, 56, 58, 61, 67, 70, 76] Social domain 3 [46, 58, 74] Knowledge and behavior 4 [63, 66, 71, 78] Physiological parameter 6 [50, 62, 63, 70, 76, 81] Musculoskeletal disorders 19 [45, 48, 51–53, 57, 60, 62, 64, 65, 67–70, 73, 74, 77, 80, 82] Dietary intake 3 [49, 63, 66] Smoking abstinence 3 [47, 49, 56] Body composition 5 [49, 62, 63, 70, 81]
Measurement of outcomes	Objective measurement 6 [54, 71, 72, 74, 79, 81] Subjective measurement 19 [45–47, 49, 53, 56, 58, 60, 61, 64–67, 75–78, 80, 83] Both objective & subjective measurement 14 [48, 50–52, 55, 57, 59, 62, 63, 68–70, 73, 82]
Other outcomes	Work-related outcomes 17 [48, 52, 54–57, 59, 61, 70, 72, 75, 77–79, 81–83] Other stated 3 [47, 49, 56]

The investigated populations mainly focused on assembly line workers (*n* = 6 [51, 57, 60, 64, 68, 69]), followed by industrial (*n* = 5 [54, 58, 63, 70, 72]), manufacturing (*n* = 5 [50, 56, 65, 79, 80]), and general factory workers (*n* = 4 [45, 62, 66, 67]). The age of the participants ranged from 17 to 65 years, with samples ranging from 24 [67] to 3,479 [79, 80] (mean: 310; median: 91), representing a total of 10,215 industrial workers with 5,606 investigated males, 3,457 females, and 1,152 workers without an exact designation of sex. Twelve studies investigated only males [47, 48, 50, 54, 55, 63, 64, 66, 69, 78, 81, 82], 6 studies only females [49, 61, 68, 72, 76, 83], and 14 both sexes [46, 51, 52, 56, 58, 59, 65, 70, 71, 73–75, 79, 80]. Twenty-seven studies examined behavioural changes [45, 46, 48, 50, 51, 53–55, 57–62, 65, 67–70, 72–76, 81–83], one study examined organisational changes [52], 10 studies examined multicomponent interventions [47, 49, 56, 63, 64, 66, 71, 77, 79, 80], and one study provided safety data [78].

Most interventions lasted three to six months (see Table 2). Seven studies [47–49, 61, 66, 77, 78] reported a post-intervention follow-up between 3 and 12 months.

The control groups in the studies received various interventions, including general treatment [46, 47, 57, 62, 65, 67, 70, 71, 73–75], educational sessions [49, 55, 66, 79–82], recommendations [48, 51, 59], recommendations with feedback [83], and ergonomic training [52]. In most studies, the control group received no interventions [45, 50, 53, 54, 56, 58, 60, 61, 63, 64, 68, 69, 72, 76–78].

The studies used various outcome measures to assess workplace health promotion, with 19 using only subjective measures [45–47, 49, 53, 56, 58, 60, 61, 64–67, 75–78, 80, 83], six only objective measures [54, 71, 72, 74, 79, 81], and 14 employing both measures [48, 50–52, 55, 57, 59, 62, 63, 68–70, 73, 82] (see Table 2). Seventeen studies examined health- and work-related outcomes [48, 52, 54–57, 59, 61, 70, 72, 75, 77–79, 81–83], 19 reported physical health-related outcomes [48, 49, 51, 55–57, 60, 62, 63, 67–70, 72–74, 77, 81, 82], 10 reported mental health-related outcomes [46, 48, 50, 52, 56, 58, 61, 67, 70, 76], and three reported social health-related outcomes [46, 58, 74]. Furthermore, six studies examined physiological parameters [50, 62, 63, 70, 76, 81], and 19 examined musculoskeletal disorder parameters [45, 48, 51–53, 57, 60, 62, 64, 65, 67–70, 73, 74, 77, 80, 82]. Additionally, four studies focused on knowledge and behaviour [63, 66, 71, 78], three on dietary intake [49, 63, 66], three on smoking abstinence [47, 49, 56], and five on body composition [49, 62, 63, 70, 81].

### Risk of bias and study quality assessment

**Fig. 2:**
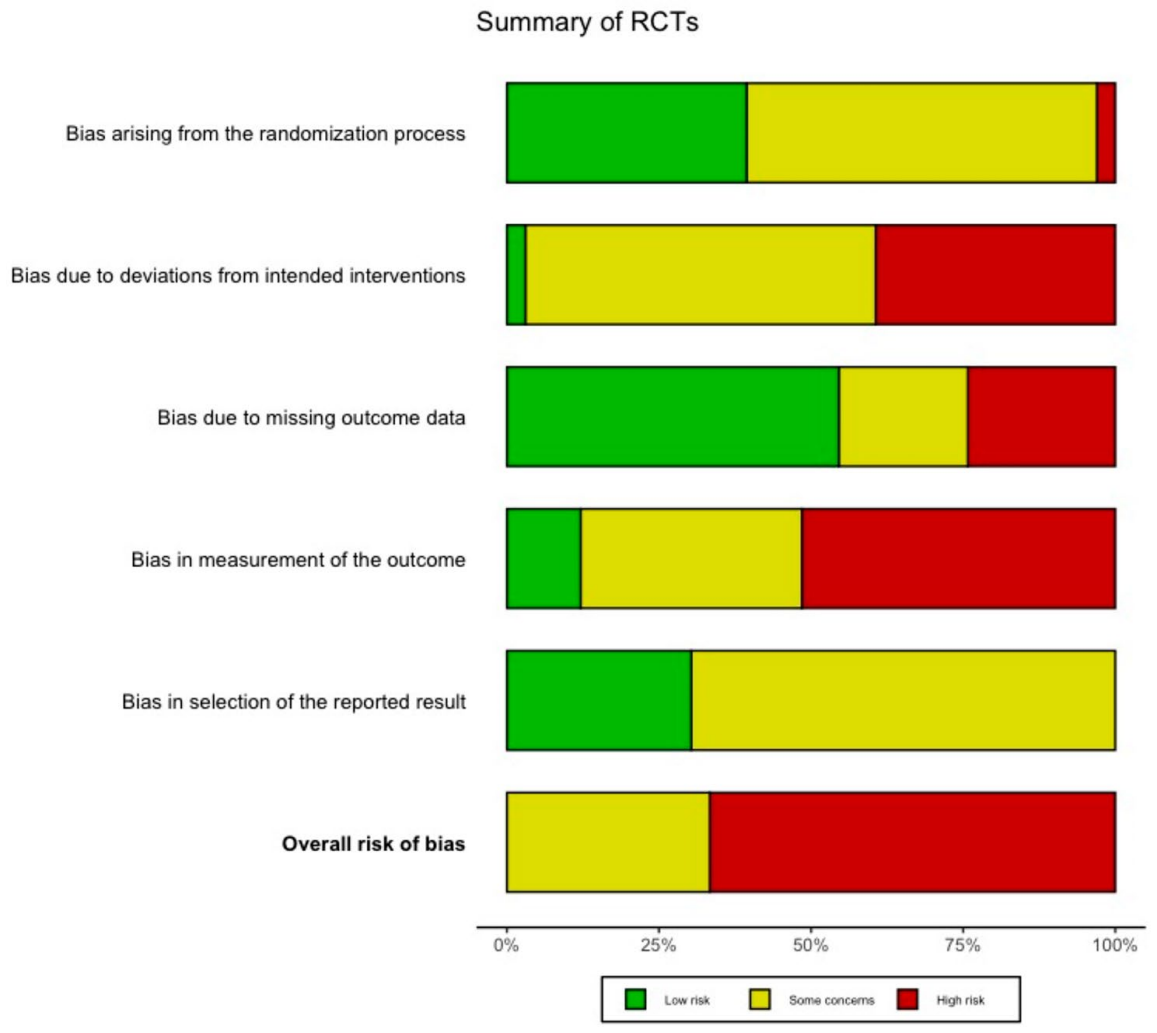
Summary of RoB 2.0 assessment for RCTs

**Fig. 3:**
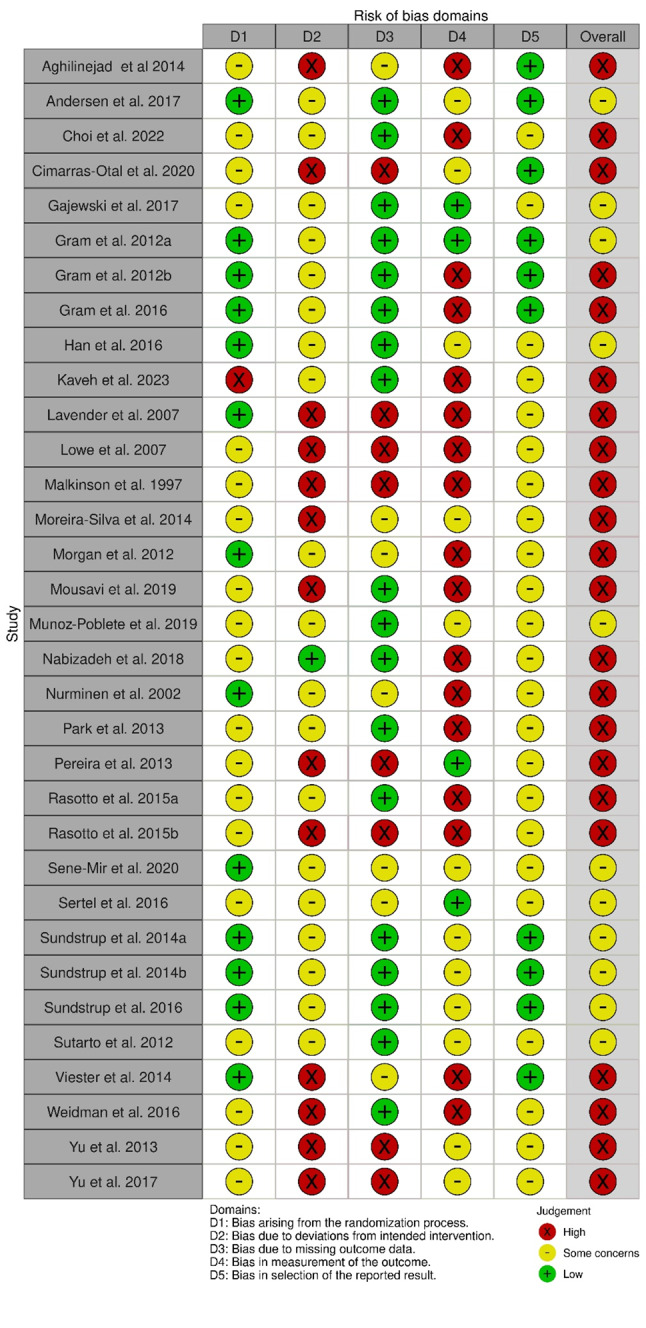
Traffic light plot for RCTs

**Fig. 4:**
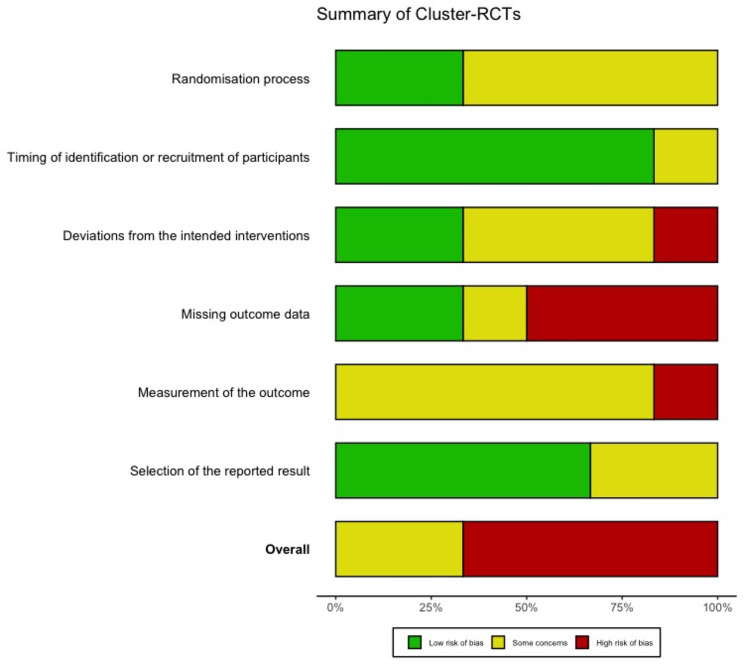
Summary of RoB 2.0 assessment for cluster-RCTs

**Fig. 5:**
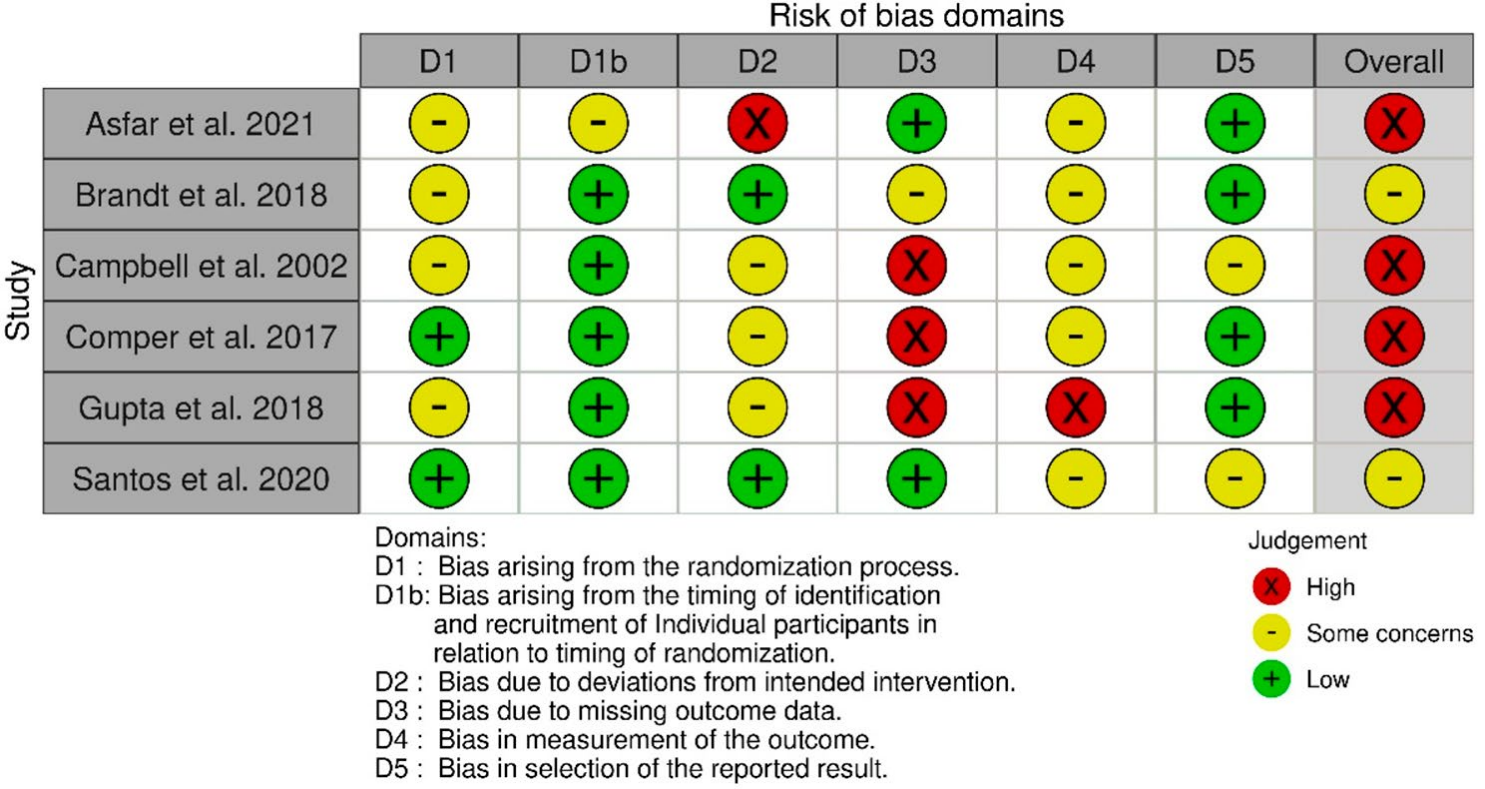
Traffic light plot for cluster-RCTs

The results of the risk of bias assessment for the RCTs are illustrated in Figs. 2, 3, 4 and 5. No studies were excluded based on their risk of bias. All relevant studies were included to provide a comprehensive overview, with quality assessment conducted to inform the analysis. Most studies (*n* = 22) have a high overall risk of bias [45, 50, 51, 53, 55, 58–64, 66–69, 77–80, 82, 83]. The main reasons for this judgment include a high risk of bias in deviations from the planned intervention, inadequate outcome data, and difficulty in measuring outcomes. The high overall risk of bias in eight studies arose from the domain measurement of the outcome [50, 55, 63, 66–68, 82, 83]. Awareness of the outcome assessors and the possible influence was considered as a high potential source of bias. The remaining articles are being judged as some overall concerns [46, 54, 57, 65, 71–76, 81]. The reasons are high risk for insufficient report deviation from predesigned intervention or missing outcome data or a lack of a pre-specified protocol, which raised concerns about selection bias. No study was judged to have a low overall risk of bias.

For the cluster-RCT, four studies were judged as high risk of bias [47, 49, 52, 56], mainly because of a high risk for missing outcome data. One study was judged with a high risk of bias due to the domain deviations from the intended interventions [47]. The other two studies were judged with some concerns [48, 70]. No study was judged to have a low risk of bias.

The methodological study quality assessments are presented in the Tables 3, 4, 5 and 6. The mean quality score for all the studies was high (71%), ranging from 39% [78] to 93% [73–75]. The corresponding scores were high for the categories’ behaviour (71%), organisational (91%) and multicomponent (71%), while safety was moderate (39%). Question 6 received a positive “yes” score across all studies. Similarly, Question 16 received a “yes” score except for the behaviour category.

**Table 3:** Characteristics and synthesis of the studies with behavioral approach using PICO scheme and quality assessment

Study	Sample size (*n*)	Population	Intervention	Control	Outcome	Score (%)	Quality
Aghilinejad et al. 2014 [45]	503	Factory workers who did not have an extra job within free time, history of fracture or major trauma, degenerative disk disease, spondylosis, spinal stenosis, neurological deficit and systemic illness or are on vacation (intervention 31 ± 5 years; control 30 ± 2 years).	First education group got lecture on LBP and related ergonomic aspects. Second education group were given a pamphlet which contained schematic diagrams reflecting the topics LBP and related ergonomy as presented orally to the first group. Third education group had a workshop discussing several aspects of LBP and ergonomics.	No intervention.	prevalence of low back pain	61	moderate
Andersen et al. 2017 [46]	66	Mainly male slaughterhouse workers working at least 30 h per week, have upper limb musculoskeletal pain of at least 3 on a scale of 0 to 10, some work disability, not having participated in either strength training or ergonomics instruction during last year (on average 45 ± 10 years).	Group-based physical exercise consisting of eight different exercises. Sessions were supervised. Load and resistance were increased according to the principle of progressive overload: Repetitions decreased over time from a maximum 20 to 8 as a standard for each exercise and load increased.	Individual ergonomic training and education based on workplace analysis and hazard prevention system.	Social climate; SF-36 (Mental health and Vitality)	82	high
Brandt et al. 2018 [48]	80	Male construction workers (intervention 34.2 ± 12.5 years; control incomplete data).	Participants received 3 workshops due to reflection of the working progress in 3 phases to improve physical exertion.	Handouts about WMSD and lifting guidelines.	Physical workload; fatigue after a typical workday; desire to influence the work; pain intensity	88	high
Choi et al. 2022 [50]	42	Male manufacturing workers who were full-time employees from a public sector, being adult (18 to 65 years) and were in active employment in manufacturing industry. Exclusion criteria were concurrent psychiatric illnesses, being a frequent user of natural environments (visiting more than 3 times a week), taking medications including hypertensive, diabetes or pain relievers (including oral hormone/corticosteroid drugs) and history of outdoor allergens in which allergic disease can exacerbate of psychological stress and discomfort (intervention 43.5 ± 7.3 years; control 45.9 ± 8.2 years).	Forest therapy.	No intervention.	Heart rate variability; stress; mood states; depression treatment, health related quality of life; cortisol level	68	high
Cimarras-Otal et al. 2020 [51]	40	Mainly male assembly line workers with chronic low back pain for at least the previous 3 month, not incapacitated in terms of job performance and owned a smartphone. Were excluded with lumbar lesion that did not allow to perform at work or undertake a physical exercise program and incompatible smartphone (on average 42.2 ± 6.2 years).	Tailored compensatory exercise, strengthen inactive muscle groups, stretching active muscles, mobilize lumbar spine and increase cardiovascular exercise. Additionally general exercise recommendations based on ACSMs approach.	General exercise recommendations based on ACSMs approach.	Lumbar disability; pain intensity; angle, bending speed; flexion-extension ratio	75	high
Gajewski et al. 2017 [54]	60	Male healthy industrial workers (on average 46.5 ± 4.5 years).	Reception of trainer-guided cognitive training (formal paper- and computer-based).	Delayed intervention but mixed with stress management and relaxation training (REL) in the first eight sessions and in the remaining 12 sessions same as intervention group.	Reaction times; Error rates; event-related potentials	50	moderate
Gram et al. 2012 [81]	67	Male construction workers with physical demanding work in including high peak demands and work for more than 20 h per week (on average 43.7 ± 10.5 years).	Participants received supervised and individually tailored aerobic capacity training and muscle strength training.	Presentation about general health promotion and usual care.	Isometric strength of different body parts; systolic and diastolic blood pressure; HDL and LDL-cholesterine; triglycerides; body weight; BMI; body fat percentage; maximal oxygen consumption;	89	high
Gram et al. 2012 [82]	102	Male construction workers with physical demanding work in including high peak demands and work for more than 20 h per week (on average 43.7 ± 10.5 years).	Participants received supervised and individually tailored aerobic capacity training and muscle strength training.	Presentation about general health promotion and usual care.	Pain intensity of different body parts; work ability; perceived physical exertion at work; productivity; sick leave	89	high
Gram et al. 2016 [55]	67	Male construction workers with physical demanding work in including high peak demands and work for more than 20 h per week (on average 43.7 ± 10.5 years).	Participants received supervised and individually tailored aerobic capacity training and muscle strength training.	Presentation about general health promotion and usual care.	Physical activity; Energy expenditure due to physical activity; Intensity of physical activity	79	high
Han et al. 2016 [57]	100	Assembly line workers with low back pain, working at least 8 h per day in a standing position and had less than 80° of the Straight leg raise (SLR) test. Participants were excluded at history of fracture and surgery, with disc herniation, acute low back pain, systemic diseases, osteoarthritis, spondylolisthesis and spondylolysis, leg length discrepancy, pregnancy and any low back pain with known causes (intervention groups 38.1 ± 6.5 and 39.7 ± 7.6 years, respectively; control 37.8 ± 8.7 years).	First intervention group have done pelvic control hamstring stretching and lumbopelvic muscle strengthening. Second group received general hamstring stretching.	Participants were told to do home program and received the handout with lumbopelvic muscle strengthening and hamstring stretching methods.	leg raise test; sit and reach test; pain intensity; workability	68	high
Kaveh et al. 2023 [58]	106	Mainly male industrial workers with more than one year of job tenure, no history of stress management education nor psychiatric treatment. Were excluded when absent from two educational sessions, not attending the pre-test or post-test sessions, withdrawal from the study or undergoing treatment by a psychiatrist due to mental illness during the intervention (intervention 35.8 ± 5.2 years; control 36.2 ± 5.9 years).	A transactional model-based education programme delivered to participants to enhance stress-coping skills. Methods were lecture, question/answer, group discussion and role play.	No intervention.	stress; spiritual well-being; perceived stress coping mechanism; social support	68	high
Lavender et al. 2007 [59]	1977	Workers with required repetitive lifting (on average 33.5 years).	Participants received the LiftTrainer™ program that was conducted by a coach using a 1-on-1 format that allowed the training to be individualized for each employee.	Video-training program.	Injury rates	46	moderate
Lowe et al. 2017 [60]	76	Assembly workers with work that have overhead processes in common (intervention 33.3 ± 8.6 years; control 37.4 ± 10.3 years).	Participants received an exercise program included resistance band strengthening movements and stretching/lengthening of the pectoralis and trapezius muscles. Progressive overload was given. Participants were encouraged to attend as many sessions per week as possible.	No intervention.	Shoulder functionality; discomfort of the shoulder; musculoskeletal symptoms	46	moderate
Malkinson et al. 1997 [61]	27	Female production workers with little formal education, based on the cognitive approach of Rational-Emotive-Behavioral Training (on average 35.3 years).	Participants received rational-emotive behavior therapy.	No intervention.	Burnout; tension; listlessness; cognitive weariness; work-home conflict	46	moderate
Moreira-Silva et al. 2014 [62]	74	Factory workers (on average 38.4 ± 7.4 years).	Participants received physical exercise focused on stretching of different body regions. Additionally, they participated in strength exercises for lower extremities. Participants were given a program to workout at home. They were asked to start recreational aerobic activities in every session.	Usual care.	Musculoskeletal pain intensity; physical activity; blood pressure; body fat; waist circumference	57	moderate
Muñoz-Poblete et al. 2019 [65]	109	Manufacturing workers with a maximum age of 40 years and asymptomatic or light musculoskeletal issues of the upper extremities with low intensity (VAS ≤ 30 mm; OCRA-Score ≥ 7.5). They were excluded with a work history lesser than one year, history of musculoskeletal trauma of the upper extremities and cardiovascular or other systemic diseases (on average 28.7 ± 5.4 years).	Participants received training sessions aiming a progressive strength training with resistance bands. In phase one shoulder stabilized muscles were focused. At least forearm muscles, shoulder rotation and elevation were added in phase 2 and 3.	Daily routine of established stretching exercises should go on. One by a physiotherapist lessoned worker supervised those exercises.	Pain intensity of different body regions	63	moderate
Nurminen et al. 2002 [83]	260	Female laundry workers with physically demanding work who were permanently employed and without contraindications for physical capacity tests (intervention 40.7 years; control 39.1 years).	Participants received feedback on their physical capacity from a physiotherapist, individual exercise prescription, counseling and worksite exercise training which involved strength training, cardiovascular exercise and stretching with progressively intensity increasing.	Participants received feedback on their physical capacity from a physiotherapist, individual exercise prescription and counseling.	Job satisfaction; workability; sick leave	68	high
Park et al. 2013 [67]	24	Factory workers diagnosed with low back pain for at least 3 months, not experienced operative interventions related to spina bifida, prolapse or spinal canal stenosis (on average 44.3 ± 5.2 years).	First intervention group received stabilization exercise for the lumbar spine, additionally to physiotherapeutic treatment. Second group have done exercises with game console Nintendo Wii.	Physiotherapeutic treatment.	Pain intensity; isometric strength; Balance ability; RAND-36 physical and mental score	54	moderate
Pereira et al. 2013 [53]	61	Garment workers who have worked in the sewing and finishing sectors of the clothing factory, have done this type of work for at least 1 year, are willing to participate in the research by signing a consent form and have never participated in a physical activity program at the workplace before. Excluded with clinical manifestations which were incompatible with the programme offered, such as a physical or mental disability and/or any pre-existing disorder that could be aggravated by participating in a physical activity program at the workplace (intervention 28.7 ± 8.8 years; control 27.8 ± 7.4 years).	Physical activity programs consisting of stretching exercises (40%), muscular endurance (40%), self-massage relaxation and massage techniques (10%) and group dynamics (10%).	No intervention.	Pain intensity	57	moderate
Rasotto et al. 2015 [69]	68	Male assembly workers at least 18 years or older who had no training specific contraindications. Excluded when participated at a structured exercise program for physical activity in the last 6 month, had diseases of the central nervous system like hemiparese, myelopathy, cerebral ataxy and muscular deformities like amputation or scoliosis and abnormalities like arthritis, that limitated movement through pain, a history of cardiovascular disease which made exercise impossible like angina or position dependable hypotonus (on average 41.1 ± 7.7 years).	Participants received tailored exercise training sessions. The first month for familization. Complaint specific exercises have been chosen. with pain mainly mobilization exercise, without pain strength exercises with light dumbbells or resistance bands carried out.	Individual daily activity has been maintained.	Pain intensity; shoulder elevation; shoulder abduction; head flexion; head extension; head rotation; head lateral inclination; handgrip strength; disability	86	high
Rasotto et al. 2015 [68]	60	Female assembly workers who had no training specific contraindications. Excluded when participated at a structured exercise program for physical activity in the last 6 month, had diseases of the central nervous system like hemiparese, myelopathy, cerebral ataxy and muscular deformities like amputation or scoliosis and abnormalities like arthritis, that limitated movement through pain, a history of cardiovascular disease which made exercise impossible like angina or position dependable hypotonus (on average 39.2 ± 6.2 years).	Participants received tailored exercise training sessions. The first month for familization. Complaint specific exercises have been chosen. with pain mainly mobilization exercise, without pain strength exercises with light dumbbells or resistance bands carried out.	No intervention.	Pain intensity; shoulder elevation; shoulder abduction; head flexion; head extension; head rotation; head lateral inclination; handgrip strength; disability	86	high
Santos et al. 2020 [70]	204	Mainly male industrial workers allocated in production sectors aged 18 to 65 years. Were excluded if they got outsourced or temporary restrictions from the medical department (intervention 34.3 ± 11.9 years; control 37.7 ± 12.9 years).	Participants received resistance exercises using progressively greater loads.	General exercise using elastic bands.	Maximum muscle strength; physical activity; fatigue; blood pressure; musculoskeletal disorders; body fat; BMI; perceived risk factors; productivity	86	high
Sertel et al. 2016 [72]	91	Female industrial workers without any disease or condition known to affect the physical performance, a history of an occupational accident and/or disease and any other injuries and conditions resulting in limited range of motion, had no reduced strength and endurance, and deformity in any of the upper extremity joints, not experienced pain and/or musculoskeletal problems or demonstrating unwillingness during the assessments and exercise programs (on average 33 ± 5.4 years).	First intervention group have done elastic bands strengthening. Second group received endurance training with repetitive movements.	No intervention.	Maximal oxygen consumption; Hand skills; handgrip strength	68	high
Sundstrup et al. 2014 [75]	66	Mainly male slaughterhouse workers currently working for at least 30 h a week, had pain intensity in the shoulder, elbow/forearm, or hand/wrist of 3 or more on a 0 to 10 visual analog scale (VAS) during the last 3 months, stated at least some work disability scoring on a 5-point scale, had no participation in resistance training during the last year or no ergonomic instruction during the last year. They were excluded with hypertension, a medical history of cardiovascular diseases, recent traumatic injury of the neck, shoulder, arm, or hand regions and pregnancy (intervention 48 ± 9 years; control 43 ± 9 years).	Participants received resistance training.	Ergonomic training (usual care).	Workability	93	high
Sundstrup et al. 2014 [73]	66	Mainly male slaughterhouse workers currently working for at least 30 h a week, had pain intensity in the shoulder, elbow/forearm, or hand/wrist of 3 or more on a 0 to 10 visual analog scale (VAS) during the last 3 months, stated at least some work disability scoring on a 5-point scale, had no participation in resistance training during the last year or no ergonomic instruction during the last year. They were excluded with hypertension, a medical history of cardiovascular diseases, recent traumatic injury of the neck, shoulder, arm, or hand regions and pregnancy (intervention 48 ± 9 years; control 43 ± 9 years).	Participants received resistance training.	Ergonomic training (usual care).	Pain intensity; disability; Shoulder rotation strength; Wrist extensor strength	93	high
Sundstrup et al. 2016 [74]	66	Mainly male slaughterhouse workers currently working for at least 30 h a week, had pain intensity in the shoulder, elbow/forearm, or hand/wrist of 3 or more on a 0 to 10 visual analog scale (VAS) during the last 3 months, stated at least some work disability scoring on a 5-point scale, had no participation in resistance training during the last year or no ergonomic instruction during the last year. They were excluded with hypertension, a medical history of cardiovascular diseases, recent traumatic injury of the neck, shoulder, arm, or hand regions and pregnancy (intervention 48 ± 9 years; control 43 ± 9 years).	Participants received resistance training.	Ergonomic training (usual care).	Handgrip strength; Fatigue for handgrip; self-related health; Pain intensity	93	high
Sutarto et al. 2012 [76]	27	Female healthy manufacturing workers with little formal education, based on the cognitive approach of Rational-Emotive-Behavioral Training (on average 35.3 years).	Participants received rational-emotive behavior therapy with biofeedback training of HRV.	No intervention.	heart rate variability; stress; depression; anxiety	50	moderate

### Behavioural (including exercise) approaches

A total of 27 studies adopting behavioural approaches in workplace health promotion programs for industrial workers were identified (Table 3). The studies included a variety of interventions, such as educational sessions, exercise programs, ergonomic initiatives, and biofeedback training.

### Very short-term interventions (< 6 weeks, *n* = 3)

Three studies with a behavioural approach used very short-term interventions. One educational study discovered that providing a lecture about low back pain and ergonomic aspects in workers from an automobile factory was beneficial in reducing the incidence of musculoskeletal disorders when compared to the control group [45]. Another study compared a three-day forest therapy to a control group and found beneficial effects on heart rate variability, stress, and mood states. Additionally, improvements in depression treatment, health-related quality of life, and cortisol levels were reported in the intervention group [50]. A study with biofeedback training was more beneficial for heart rate variability, stress, depression, and anxiety than the control group [76].

### Short-term interventions (6–11 weeks, *n* = 9)

Nine studies have utilised short interventions. In one study educational sessions about stress management were beneficial on stress, spiritual well-being, perceived stress-coping mechanisms, and social support compared to a wait-list control group [58]. The other eight studies compared exercise programs. One study used an elastic band training on healthy female workers, which resulted in improved handgrip strength but no change in VO2 max and on hand skills as a work-related outcome compared to a control group [72]. Seven studies focused on industrial workers with acute or chronic low back pain and compared various exercise programs such as group resistance training [46, 73–75], work-adapted exercises [51], a hamstring stretch program [57], and lumbar stabilisation and Nintendo Wii exercise program [67] to control groups with recommendations [51], personalised ergonomic training [46, 73–75], a generalised treatment [67], and a handout and home training [57]. Mixed effects on physical parameters were reported (improvements in leg raise and sit and reach test strength [57]; increased handgrip strength [74]; shoulder and wrist strength [73]), improved time to fatigue for handgrip [74] and flexibility [51] but not on isometric strength or balance ability [67]). Additionally, mixed effects have been reported regarding mental health (from the SF-36) with beneficial [67] and no effects [46]. However, improvements were found in self-related health [74], social climate and vitality [46], pain intensity [57, 67, 73, 74], pain interference [51], workability [57, 75], and disability [73].

### Mid-term interventions (3–6 months, *n* = 12)

Two of twelve studies used an educational approach in healthy industrial workers. A stress management program led to beneficial changes in burnout, listlessness, cognitive weariness and work-home conflict as work-related outcomes but not in tension against a control group [61]. Ergonomic initiatives have led to beneficial effects for heavy lifting and fatigue after a typical workday and an increased desire to influence their work but not on pain compared to a control group, who got recommendations [48]. Also cognitive training induced improvements in reaction times, error rates, and event-related potentials compared to a control group [54]. Moreover, a shoulder exercise program benefited shoulder functionality but did not improve musculoskeletal symptoms on assembly line workers [60], whereas an upper-body resistance training program with elastic bands decreased pain intensity [65]. A tailored mobilisation program improved range of motion, handgrip strength, pain intensity, and disability [68]. Stretching exercises lead to beneficial effects on pain intensity [53, 62], while no effects were observed on self-reported physical activity, blood pressure, waist circumference, or body fat [62]. A tailored aerobic and strength training program on construction workers [55, 81, 82] increased VO2max [81] but did not change other parameters compared to a control group with educational sessions (isometric strength [81], perceived physical exertion [82], blood pressure, lipid parameters, BMI [81], pain intensity, workability, productivity, sick leave [82]). Furthermore, after this treatment, a decrease in objectively measured physical activity during weekdays and leisure time was reported compared to the control group [55]. In a study with progressive resistance training no effects were observed on any outcome (maximum muscle strength, self-reported physical activity, perceived fatigue, blood pressure, musculoskeletal complaints, body fat, BMI, perceived risk factors, or productivity) compared to a control group who performed general exercise using elastic bands [70].

### Long-term interventions (> 6 months, *n* = 3)

A long-term intervention using strengthening, endurance, and stretching exercises on female industrial workers did not lead to beneficial effects on job satisfaction, workability, or sick leave compared to controls which received recommendations and feedback [83]. A tailored mobilisation and strengthening program on manufacturing workers improved range of motion, handgrip strength, pain intensity, and disability [69], whereas a biofeedback training study found no beneficial effects on injury rates compared to a control group [59].

### Organisational interventions

Only one study implemented ergonomic training followed by a 12-month job rotation at the organisational level (Table 4). However, no improvements were found compared to a control group that received ergonomic training (need for recovery, quality of life, musculoskeletal disorders, prevalence of pain, sick leave, productivity) [52].

**Table 4:** Characteristics and synthesis of the studies with organisational approach using PICO scheme and quality assessment

Study	Sample size (*n*)	Population	Intervention	Control	Outcome	Score (%)	Quality
Comper et al. 2017 [52]	266	Mainly female manufacturing workers arranged in a manufacturing cellular and serial layout with jobs or tasks with different biomechanical demands and levels of risk for musculoskeletal disorders (intervention 28.4 ± 7.8 years; control 32.5 ± 9.0 years).	Ergonomic training and additional job rotation were performed.	Only ergonomic training.	Need for recovery; quality of life; musculoskeletal disorders; prevalence of pain; sick leave; productivity	91	high

### Multicomponent approaches

Ten studies have implemented a multicomponent approach of different durations [47, 49, 56, 63, 64, 66, 71, 77, 79, 80] (Table 5). These studies combined interventions such as educational training, behavioural approaches, exercise programs, and nutritional education.

**Table 5:** Characteristics and synthesis of the multicomponent-studies using PICO scheme and quality assessment

Study	Sample size (*n*)	Population	Intervention	Control	Outcome	Score (%)	Quality
Asfar et al. 2020 [47]	134	Male construction workers ≥ 18 years old who were hispanic or latino, smoking ≥ 5 cigarettes per day for the past year, were interested in making a quit attempt in the next 30 days, having access to a telephone and not planning to move in the next 6 months. They were excluded if having a contraindication to nicotine replacement treatment (NRT; e.g. recent myocardial infarction, history of serious arrhytmias) or inability to understand consent procedures (intervention 40.9 ± 1.5 years; control 38.2 ± 1.4 years).	Based on social cognitive theory participants received one culturally adapted face-to-face counseling session, followed by two brief phone calls, 6 weeks of NRT, fax referral to Florida quit line (four brief phone conseling sessions; two weeks of free NRT), informative handouts about the Florida quit line and benefits of quitting smoking.	Only 6 weeks of NRT, fax referral to Florida quit line (four brief phone conseling sessions; two weeks of free NRT) and informative handouts about the Florida quit line.	smoking abstinence; feasibility and acceptance of the intervention	73	high
Campbell et al. 2002 [49]	649	Female blue-collar workers over 18 years old, speaking English or Spanish from small to medium-size blue collar industry which employing a majority of women. It had no systematic health promotion program currently in place and no plans for immediate plant closure. Excluded were factories where not enough women or too many employees were shown, the plant closed or about to close, already had a comprehensive health promotion program, was not the right industry or county, had no permanent employees, had a wrong address or duplicate listing and did not have authority at the worksite to agree to a program (no mean age reported).	Individualized computer-tailored “women’s magazines”; a natural helpers intervention that trained women in the workplace to diffuse information and a health education to support healthy behavior changes.	Delayed intervention worksites were offered a menu of possible health education sessions for their employees on topics not directly related to study objectives; after 6 month same tailored magazines as intervention but no natural helpers program.	Consumption of fruits and vegetables; smoking abstinence; BMI, cancer screening; flexibility; fat score; physical activity	43	moderate
Gupta et al. 2018 [56]	415	Manufacturing workers from workplaces with a minimum of 100 workers and team-based physical work with cooperative relations between hierarchy levels. Teams required formal work groups and individuals had to have upon 20 h work time per week and study consent (on average 44.1 ± 10.5 years).	Workshops were conducted at different levels of the organization to enhance productivity, wellbeing, and health and safety. At the group level, participants attended three workshops. The first workshop focused on visual mapping, teaching participants to identify factors that positively or negatively affect workability, such as demands and resources. The second workshop centered on action planning, where participants translated their learnings into concrete plans displayed on whiteboards. The effectiveness of these plans in supporting productivity, wellbeing, product quality, and cost effectiveness was evaluated. The third workshop provided an opportunity for workers and supervisors to exchange ideas and offered additional options like individual visual mapping talks and ergonomic workshops. At the leadership level, ambassador workshops were held to disseminate knowledge on promoting health and safety among workers. These workshops aimed to foster a culture of well-being and safety throughout the organization.	No intervention.	Need for recovery; Workability; Productivity; Physical work demands; Physical resources; leisure-time physical activity; Well-being; Mental health; smoking abstinence	89	high
Morgan et al. 2012 [63]	110	Male industrial shift workers who were overweight or obese (BMI between 25 and 40 kg/m^2^), aged 18–65. Excluded at history of major medical problems such as heart disease in the last 5 years, diabetes, orthopaedic or joint problems that would be a barrier to physical activity, recent weight loss of ≥ 4.5 kg or taking medications that might affect body weight (on average 44.4 ± 8.6 years).	Behaviour change strategies consisting of information session, handbook, study website, website tutorial and user guide. 7 individualized dietary feedback sheets and group-based financial incentive pedometer were given.	No intervention.	Body weight; waist circumference; BMI; blood pressure; resting heart rate; Physical activity; Dietary; Physical activity cognitions; Healthy eating practices; Dietary state of change (SOC)	75	high
Mousavi et al. 2019 [64]	100	Male production line workers with at least 1 year of work experience and standing for at least 3 h per day at work. Participants were excluded with any reports of lower limb or back injury such as fracture, dislocation, soft tissue lesions (during the previous 6 months), any history of surgery in the muscles, joints, or bones in the lower back and lower extremities, musculoskeletal diseases and neuromuscular disorders such as joint rheumatism, myopathy or neuropathy, foot deformity including raised or flattened arches or hallux valgus, and any vascular problems in the lower extremities (on average 35.9 years).	First group got custom-made insoles and lower limb exercises. Second intervention group had insoles only, and third group had lower limb exercises only.	No intervention.	Musculoskeletal disorders; discomfort level in back, thigh, knee, leg and foot.	71	high
Nabizadeh et al. 2018 [66]	210	Male cement factory workers who did not present a history of, or current, physical symptoms of serious neurological, cardiovascular, renal, hepatic, endocrine, metabolic or gastrointestinal disease and previous pharmacological treatment, according to the medical reports available at the factory (intervention groups 34.6 ± 4.5 and 33.8 ± 4.9 years, respectively; control 34.7 ± 4.8 years).	Participants received educational interventions based on the Protection Motivation Theory (PMT). Face-to-face group had multimodal lectures with powerpoint presentations, discussion, questions and answers, individual counselling sessions and educational pamphlets and booklets. Indirect group received recently designed educational content through pamphlets and booklets.	No intervention.	Motivation; knowledge; intention; consumption of Vitamin C and E	64	moderate
Sene-Mir et al. 2020 [71]	61	Mainly female blue-collar workers ≥ 18 years old who not suffer any chronic bone, muscle, or joint disease in the trunk, and/or chronic or acute pain diagnosed by a specialist. Not suffer any chronic or acute knee joint disease diagnosed by a specialist (no age described).	Received intervention which contained systematic self-observation, hetero-observational Feedback as well as feedforward and intrinsic feedback.	Standard manual material handling training.	Knowledge of working postures	68	high
Viester et al. 2014 [77]	314	Construction and production workers without sick leave within last 4 weeks before intervention (on average 46.6 ± 9.7 years).	Participants received training to improve lifestyle including individual information, personal or phone contacts, exercises and material tailored to body weight, physical activity and motivation of lifestyle change. They got a periodic review of knowledge about healthy lifestyle and will to change.	Maintainance of daily activities.	Physical functioning; musculoskeletal disorders; workability; sick leave; work-related vitality; performance	86	high
Yu et al. 2013 [80]	3479	Mainly male manufacturing workers employed at medium-size industrial companies (with employees from 300 to 2,000) that should be matched with another factory by industry and production processes, with a less than 30% turn-over rate of workers in 1 year. Participants: were frontline workers and being employed in the current factory for at least 12 months. They were excluded if employed in administration, design and logistics or are illiterate and seasonal migrant workers (intervention 29.1 ± 7.3 years; controls 28.9 ± 7.4 and 28.3 ± 7.1 years, respectively).	Participatory training of learning successful examples from other workplaces. Program includes personal protective equipment usage, stretching and strengthening exercise and games including ergonomic and materials handling.	Didactic training of only a short presentation, without group discussions, games or workplace visits.	Musculoskeletal disorders	75	high
Yu et al. 2017 [79]	3479	Mainly male manufacturing workers employed at medium-size industrial companies (with employees from 300 to 2,000) that should be matched with another factory by industry and production processes, with a less than 30% turn-over rate of workers in 1 year. Participants: were frontline workers and being employed in the current factory for at least 12 months. They were excluded if employed in administration, design and logistics or are illiterate and seasonal migrant workers (intervention 29.1 ± 7.3 years; controls 28.9 ± 7.4 and 28.3 ± 7.1 years, respectively).	Participatory training of learning successful examples from other workplaces. Program includes personal protective equipment usage, stretching and strengthening exercise and games including ergonomic and materials handling.	Didactic training of only a short presentation, without group discussions, games or workplace visits.	Incident rates of accidental injury	68	high

### Very short-term interventions (< 6 weeks, *n* = 2)

Two studies included educational training with the addition of behavioural approaches. A multi-component program on quitting smoking from construction workers had no beneficial effect on smoking abstinence but got high feasibility and an acceptable rate against a control group with recommendations [47], whereas a self-observation and feedback program on blue-collar workers compared to a control group with general treatment was beneficial on knowledge of working postures [71].

### Short-term interventions (6–11 weeks, *n* = 2)

Two studies on factory workers used custom insoles and lower limb exercise training in a three-group randomised control design showed beneficial effects on musculoskeletal disorders in favour of the group that received both training and insoles [64]. A nutrition education program benefited workers’ motivation, knowledge, intention, and consumption of specific vitamins more than a control group who received educational pamphlets [66].

### Mid-term interventions (3–6 months, *n* = 3)

A program focusing on weight loss was beneficial for physical activity and physical activity-related cognition, physiological parameters (resting heart rate, blood pressure), body compositions (BMI, weight loss, and waist circumference), and some parts of dietary intake (increased consumption of fruits and sweetened beverages, but non-beneficial on vegetable and alcohol consumption) compared to a wait-list control group [63]. A tailored program on female workers increased consumption of fruits and vegetables but led not to smoking abstinence, lower BMI, and cancer screening. Additionally, improvements in flexibility and dietary fat scores were reported to be beneficial after six months but not after a follow-up of 18 months. An increase in physical activity in the intervention group was reported, but the difference was not statistically significant compared to control group with educational sessions [49]. One intervention program on construction workers focusing on preventing and reducing overweight and musculoskeletal symptoms was non-beneficial on physical functioning, musculoskeletal disorders, workability, sickness absence, work-related vitality and performance against a control group [77].

#### Long-term interventions (> 6 months, *n* = 3)

In manufacturing workers educational sessions, strength and stretching training, and games reduces work-related injuries [79] and musculoskeletal disorders in specific body parts compared to a control group with educational sessions [80]. In contrast, different workshops have no beneficial effects in industrial workers compared to a control group (leisure-time physical activity, physical resources, need for recovery, well-being, mental health, smoking abstinence, workability, physical work demands, productivity, or employees’ appraisal of intervention activities) [56].

### Safety interventions

One intervention study on drywall-finishing workers was conducted to improve safety outcomes through education. Positive effects were found on self-efficacy and trust in technology, but not in health knowledge, perceived health risk, and trust in the organization when compared to the control group (Table 6). For the parameter readiness to adopt the tool, beneficial effects were reported at the end of the intervention but not at the follow-up [78].

**Table 6:** Characteristics and synthesis of the safety interventions studies using PICO scheme and quality assessment

Study	Sample size (*n*)	Population	Intervention	Control	Outcome	Score (%)	Quality
Weidmann et al. 2016 [78]	40	Male drywall-finishing workers.	Didactic and interactive training, information about dust material composition, health effects of dust exposure, usefulness of ventilated sanders. Cues to action wearing hard-hat stickers and t-shirts. Improving worker self-efficacy and trust in technology at trying and testing the new technology.	No intervention.	Health knowledge; self-efficacy; Perceived risk to health; trust in Technology; trust in organization; adoption readiness.	39	moderate

## Discussion

In total 39 RCTs were included in this systematic review on the effectiveness of workplace-related health interventions in industrial workers. The majority of studies (*n* = 27) adopted behavioural interventions. Among them, most studies used exercise programs, followed by educational approaches. Ten studies employed multicomponent interventions with combinations of educational interventions and behavioural or organisational approaches, one opted for an organisational approach, and one study focused on safety-related outcomes. In most studies, there was a high overall risk of bias, necessitating a cautious interpretation of all results.

### Intervention characteristics

Most behavioural studies used an exercise program, while others employed an educational approach. Exercising seems to be a promising tool for health-related outcomes in musculoskeletal [29, 30, 84] and cardiometabolic disorders [31] as well as on mental health outcomes [3, 85]. Educational programs might have a positive impact on mental health [30, 86], but other health-related outcomes, such as musculoskeletal disorders, might not profit [29]. Nevertheless, a positive impact of educational strategies on work-related outcomes, as demonstrated in other contexts with shift workers [87], emphasizes their importance and warrants additional research.

Only one study used an organisational approach in form of job rotation. This is somewhat surprising as recommendations for adding organisational components to improve health-related outcomes were given [88]. The potential advantages of such interventions have yet to be thoroughly explored [9, 43, 89].

In studies on multicomponent approaches, educational programs combined with behavioural or organisational approaches were frequently used. However, inconclusive effects were reported on health-related outcomes in workplace health promotion [29, 90].

A single study was conducted to enhance safety outcomes, underscoring the need for additional research. Health-promoting behaviours might be associated with safety-related outcomes [91].

### Effectiveness on health-related outcomes

The findings from the current review revealed several studies demonstrating the effectiveness of workplace intervention in improving specific aspects of health. Mixed effects were found in the studies that examined physical outcomes. Strength outcomes were generally beneficially impacted by workplace interventions. This is consistent with previously reported findings [29, 92]. Conversely, the effects on aerobic capacity varied. Whilst other research found beneficial effects on peak oxygen consumption [93], we found no beneficial effect. An explanation for this discrepancy might be the limited number of studies incorporated in our review. In one study using objectively measured physical activity, a decrease of physical activity was found, contradicting prior research [94–96]. A systematic review and meta-analysis of workplace health promotion programs among men reported nearly half of the included studies showed improvements in physical activity outcomes [97]. The potential differences may be attributed to the fact that physical activity needs to be considered holistically to counteract a possible physical activity paradox and ensure health benefits [98].

In line with other reviews and meta-analyses, our results showed beneficial effects of workplace health promotion on mental outcomes [30, 86, 95, 99]. Regarding physiological parameters, we observed no consistent trends. While other reviews suggested that workplace health promotion affects biological risk factors [94] or enhances cardiometabolic markers [31], our findings did not provide a clear conclusion. However, a systematic review of reviews reported inconsistent findings regarding metabolic risk factors [100].

Furthermore, only a few studies showed beneficial effects on social parameters. Research emphasises the relevance of detecting these outcomes [96, 101, 102].

Regarding parameters related to musculoskeletal disorders, most studies indicated a beneficial effect of workplace interventions, regardless of duration and intervention characteristics. This finding aligns with previous research on workers with physically demanding jobs [1, 29, 30]. One systematic review of longitudinal studies showed the multifactorial etiologic of musculoskeletal disorders and identified psychosocial workplace factors associated with the risk and progression of musculoskeletal disorders [103].

Our findings indicate limited beneficial effects on body composition, as well as in interventions that focus on that topic. This somewhat contrasts beneficial outcomes reported in other research [30, 88, 90, 92, 95, 97]. These disparities may arise from the different populations. Thus, industrial workers’ body composition is a significant concern [104], necessitating different interventions and strategies. In contrast, beneficial effects on dietary outcomes were identified, particularly in the context of multicomponent studies. This aligns with prior research and underscores the significance of these findings [30, 94, 95, 105].

Our findings suggest that workplace health promotion initiatives do not significantly impact on smoking cessation rates, which is in line with the systematic review and meta-analysis from Bezzina and colleagues [97]. This contradicts the findings of a meta-analysis on general workers [106]. Nevertheless, the authors concluded that while interventions are effective in stopping smoking, the absolute number of workers who successfully quit is minimal [106], which aligns with our results.

We found heterogeneous results for work-related outcomes. This is possibly a result of the wide range and complex construct to measure work-related outcomes [14]. However, other reviews suggest the potential impact on work-related outcomes through workplace health promotion [9, 14].

Consequently, there is some potential for workplace health interventions but their effectiveness remains unclear. Additionally, it is unclear whether particular types of interventions, such as behaviour or organisational interventions, are more effective than others. Similar to previous systematic reviews on workplace health promotion, it remains challenging to establish firm recommendations about the effectiveness of workplace interventions or identify the types of interventions that demonstrate the most promise [9, 107–110].

### Population- and workplace-specific needs

In the industrial sector, sex or gender-specific needs and health hazards have to be considered [27, 111]. Most industrial workers in this review were males, so workplace health promotion interventions might be needed to favour this group. Moreover, a wide variety of industrial tasks [112] with different work paces [113], environments [114], physical demands [115, 116], and shift work [117] can impact health-related factors as possible cofounders. Also, the different health conditions of the participants demonstrate the necessity to focus on their different characteristics. Several studies have focused on industrial workers with at least one health risk. This might be an appropriate representation of industrial workers considering the major concerns related to musculoskeletal disorders [1, 9], metabolic syndrome [118], and mental health [43]. Focusing on the concerns of industrial workers could be an effective approach to developing more targeted and specialised programs [119]. This is particularly important when considering the effectiveness and transferability of interventions in lower-income countries or countries with fewer medical conditions [120]. Additionally, a systematic review reported the factors of economic, management support, intervention concepts, data collection, and resources and commitment to intervention influencing workplace health promotion [121]. Therefore, healthy leadership might be a principle which should be considered [101]. Furthermore, another systematic review reported barriers to workplace health promotion, such as time, a busy lifestyle, and lack of motivation [92]. Addressing needs with tailored activities might lead to a high participation of workplace health promotion programs [28]. Our mixed findings underscore the importance of tailoring interventions based on duration for effective implementation in an occupational health context.

Most of the studies lasted 3–6 months, which may be necessary for behavioural modification. Shorter durations may not be sufficient, as changes depend on the frequency and direction of the modifications that individuals consider [122].

This review has shown that long-term intervention effects were assessed in only a few of the studies. Moreover, many studies have not provided sufficient information regarding the follow-up duration. Only seven of the included studies had a true post-intervention follow-up.

### Risk of bias and methodological quality of studies

The high overall risk of bias in this review is consistent with findings from others [13, 31, 105]. The main sources for a high risk of bias resulted from high risks for deviations from the planned intervention, inadequate outcome data assessment, difficulty in measuring outcomes, awareness of the outcome assessor, insufficient report deviation from predesigned intervention, or a lack of a pre-specified protocol. These risks can influence the results of the individual studies in terms of an overestimation or underestimation of the intervention’s true effect [123, 124]. Future studies should prioritise the development of well-designed study protocols and enhance the transparency of data. Additionally, data collection should be conducted using valid measurement methods. Further improvements may the blinding of researchers to improve objectivity and rigour in the research process. By addressing these sources of bias, future research can provide more reliable and valid results, ultimately contributing to better evidence-based practices.

As most of the reviewed studies only relied on self-reported outcomes the measurement of health-related outcomes should be considered in future work. Therefore, certain parameters can be assessed by objective measurements collected from the industry or other sources [125]. Additionally, using single items to assess health-related outcomes increases the risk of response bias and limited possible comparisons between studies.

Numerous interventions included multiple heterogeneous components. Consequently, it is not possible to attribute the effectiveness of a particular intervention to one specific intervention component.

Some studies have reported long-term outcomes. However, the majority of studies have not used an intention-to-treat analyses or failed to report this in their publication. Thus, the generalizability of the reported results is uncertain, as it is unclear whether they exclusively apply to individuals who completed all the assessments. Additionally, the findings may have been influenced by selection bias.

## Strengths of this review

This review provides a detailed analysis of workplace health promotion interventions among industrial workers. A narrative synthesis was selected, given the study objectives and high levels of heterogeneity. A strength of this review is that it reports workplace health promotion interventions and their beneficial or non-beneficial effects on health- and work-related outcomes, which limits potential bias. Not limiting the conditions of the population to specific health conditions has shown its applicability to society. Risk of bias and a detailed quality assessment was performed for each included study.

### Recommendations for future research

This systematic review highlights several areas for future research to improve the effectiveness and reliability of workplace health promotion programs for industrial workers. One major consideration is the high heterogeneity in the parameters and interventions across the included studies. This variability can be attributed to differences in implementation fidelity, participant characteristics, and the specific context in which the interventions were applied. For instance, variations in intervention delivery, duration, intensity, and the baseline health status of participants could all contribute to the observed differences in effectiveness. However, only published studies were included. We need to acknowledge the possibility of publication bias, which may impact the findings.

Future research should include only valid and reliable measures of health-related outcomes. Furthermore, interventions may focus on improving the specific health characteristics of included industrial workers. In addition, work and leisure time, especially physical parameters, should be measured and, where possible, should be used with objective measurements. Furthermore, the issue of high drop-out rates reported in several studies is a concern that requires further investigation. To address this challenge, future research should focus on the feasibility and acceptance of different types of interventions. This approach will allow researchers and policymakers to determine the most suitable interventions for specific participants and organisations.

Future research should evaluate health-related outcomes at the end of the intervention and at regular follow-ups to determine whether any beneficial effects can be maintained, particularly for behavioural approaches. Furthermore, conducting long-term studies is essential to capture the impact of interventions throughout a more extended period of time. This might lead to a human-centred approach to promoting workplace health initiatives and principles such as the Goldilocks principle [126], which could result in the effective implementation of interventions. Despite recent research, there remains a need for well-designed studies to thoroughly assess the effectiveness of workplace health promotion interventions on health outcomes. Higher-quality studies are also necessary to enhance evidence transparency and support meta-analyses and study weighting [127]. Utilising randomisation, blinding techniques, and conducting predefined subgroup analyses can improve future research.

## Conclusions

This systematic review provides an extensive overview on workplace interventions and their impact on industrial workers’ health-related outcomes. Although the included studies provide evidence that workplace health promotion can be effective, the overall results are inconclusive. Beyond that, a high risk of bias for certain domains and a high overall risk of bias was revealed, and several methodological limitations in the current evidence-based research are given. The heterogeneity of interventions should be recognised in terms of general conclusions, and further research is needed to determine which elements are most likely to increase efficacy and adoption within the workplace setting. The specific health characteristics and conditions of industrial workers are important factors that must be considered. Consequently, drawing solid conclusions regarding the effectiveness of workplace interventions among industrial workers remain challenging. However, given the positive outcomes of some related studies, there is a strong need for implementing workplace interventions in industrial workers on the one hand, and for continuing research efforts to support national and global public health policies to improve industrial workers’ health on the other hand.

## Data Availability

The datasets used and analysed during the review are available from the corresponding author on reasonable quest.

## Abbreviations

BMI: Body mass index
PRISMA: Preferred Reporting Items for Systematic Reviews and Meta-Analyses
RCT: Randomised controlled trials
